# Prolonged 14-day continuous infusion of high-dose ifosfamide for patients with relapsed and refractory high-grade osteosarcoma: a retrospective multicentre cohort study

**DOI:** 10.1186/s12885-024-12498-x

**Published:** 2024-06-19

**Authors:** Elisa Tirtei, Anna Campello, Veronica Sciannameo, Sebastian Dorin Asaftei, Cristina Meazza, Giovanna Sironi, Alessandra Longhi, Toni Ibrahim, Angela Tamburini, Luca Coccoli, Fanj Crocco, Celeste Cagnazzo, Elvira De Luna, Paola Quarello, Paola Berchialla, Franca Fagioli

**Affiliations:** 1 Paediatric Onco-Hematology, Stem Cell Transplantation and Cellular Therapy Division, Regina Margherita Children’s Hospital, Piazza Polonia 94, Turin, 10126 Italy; 2 Department of Public Health and Paediatrics, University of Turin, Turin, Italy; 3 Centre for Biostatistics, Epidemiology and Public Health, Department of Clinical and Biological Sciences, University of Turin, Regione Gonzole 10, Orbassano, 10043 Italy; 4 Paediatric Oncology Unit, Fondazione IRCCS, Istituto Nazionale dei Tumori, Milan, Italy; 5 Osteoncology, Bone and Soft Tissue Sarcomas and Innovative Therapies, IRCCS Istituto Ortopedico Rizzoli, Bologna, Italy; 6 Department of Paediatric Haematology-Oncology, Meyer Children’s Hospital IRCCS, Florence, Italy; 7 Pediatric Oncology-Hematology Unit, Stem Cell Transplantation and EURACAN Hub Center Unit, S. Chiara Hospital, AOUP, Pisa, Italy; 8 Paediatrics Division, Department of Health Sciences, AOU Maggiore della Carità di Novara, Piemonte Orientale University, Novara, Italy

**Keywords:** Osteosarcoma, Chemotherapy, Ifosfamide, Relapse

## Abstract

**Background:**

The prognosis of patients with Relapsed/Refractory Osteosarcoma (R/R OS) remains dismal without an agreement on systemic therapy. The use of High-Dose Ifosfamide (14 g/sqm) with an external pump in outpatient setting (14-IFO) in R/R OS patients is limited. This study represents the first retrospective cohort analysis focused on evaluating the activity and toxicity of 14-IFO in this setting.

**Patients and methods:**

The study investigated 14-IFO activity, in terms of tumour response according to RECIST 1.1 criteria, as well as survival rates and toxicity, according to CTCAE v.5.

**Results:**

The trial enrolled 26 patients with R/R OS. The Overall Response Rate (ORR) and Disease Control Rate (DCR) obtained was 23% and 57.5%, respectively. Patients with relapsed OS showed a higher ORR (45%) and DCR (82%) compared to refractory patients, irrespective of the number of prior treatment lines received. The achievement of disease control with 14-IFO administration enabled 27% of patients to undergo new local treatment. Four-month Progression-Free Survival (PFS) was 54% for all patients and 82% for the relapsed OS sub-group. Median Overall Survival (OSurv) was 13.7 months, with 1-year OSurv of 51% for all patients and 71% for relapsed patients. Age over 18 years and the presence of refractory disease were identified as negative prognostic factors for this patient cohort. A total of 101 cycles were evaluated for toxic assessment, demonstrating a tolerable profile without grade 3–4 non-haematological toxicities.

**Conclusions:**

14-IFO should be considered a viable treatment option for R/R OS, particularly due to its well tolerated toxicity profile and the potential for home-administration, which can improve patient quality of life without compromising efficacy.

**Supplementary Information:**

The online version contains supplementary material available at 10.1186/s12885-024-12498-x.

## Introduction

Osteosarcoma (OS), a rare and aggressive mesenchymal tumour, is the most common primary malignant bone tumour, predominantly afflicting individuals in the second decade of life, particularly adolescents and young adults [1, 2]. Despite the introduction of a first-line multidisciplinary treatment approach improved the 5-year Overall Survival (OSurv) from 10% to 60–70% [3–6], there has been a notable absence of further improvements in cure rates over the past four decades [7, 8] and approximately 30% of patients with OS experience local or systemic recurrence [7–9].

Treatment of patients with a recurrent or refractory OS (R/R OS) remains an unmet clinical need, as evidenced by a 5-year OSurv rate of less than 30% [2, 9–11] and a lack of consensus regarding an effective systemic treatment [2, 7].

Prognostic factors influencing the survival of patients with R/R OS include the length of the relapse-free interval (RFI), the site of recurrence, and the feasibility of a new complete surgical remission, although the latter remains achievable for only few patients [1, 2, 10]. Patients with inoperable lesions receive systemic treatment with the aim to improve their Disease Control Rate (DCR) and prolong survival [10, 11], nonetheless the benefit of chemotherapy for these patients is still debated [2, 10–11].

Ifosfamide is an alkylating chemotherapy agent that has been widely used in R/R OS treatment [12–18], either alone or in combination with other agents (Table 1), demonstrating a role in improving both Disease Control Rate (DCR) and Progression Free Survival (PFS) [12–19].

**Table 1:** List of published chemotherapy regimens for patients with R/R OS

	Total pts enrolled (n°)	OS pts enrolled (n°)	OS Disease Status	Drugs and Schedule	In/Outpatient	PFS or EFS reported for OS pts	OSurv reported for OS pts	OS ORR (CR + PR)	OS DCR (CR + PR + SD)
Harris et al., 1995	63	63	- Metastatic and/or unresectable primary OS (stratum 1: 33 pts) - Relapsed OS (stratum 2: 30 pts)	IFO: 2,400 mg/sqm/day x 5 days	NR	NR	NR	- Stratum 1: 27,3% (1CR + 8PR) - Stratum 2: 10% (1 CR + 2 PR)	- Stratum 1: 72,7% (1 CR + 8 PR + 4 mixed + 11 NR/SD) - Stratum 2: 53,3% (1 CR + 2 PR + 1 mixed + 12 NR/SD)
Berrak et al., 2005	16	16	Previously treated pediatric patients with locally recurrent and/or metastatic osteosarcoma	IFO: 2 g/sqm/dose administered intravenously at 12 hr interval x 7 days = total dose 14g/sqm	Inpatient	15-month EFS: 43,7%	15 month OSurv: 44 ± 12,4%	− 62,5% (6 CR + 4 PR)	− 62,5% (6 CR + 4 PR + 0 SD)
Patel et al., 1997	74	19	NR	IFO: 14 g/sqm (bolus or continuos infusion)	Inpatient	NR	NR	-42%	NR
Verschoor et al., 2020	62	62	Recurrent/metastatic	IFO 5 g/sqm as bolus infusion (26 patients) or IFO 3 g/sqm x 3 days continuos (36 patients)	NR	Median PFS: 2,6 months (whole cohort) Median PFS: 2,1 months (dose 5g/sqm) Median PFS: 3,8 months (dose 9g/sqm) 4-month PFS: 12% (dose 5g/sqm) 4-month PFS: 44% (dose 9g/sqm)	Median OSurv: 9,1 months (whole cohort) Median OSurv: 6,7 months (dose 5g/sqm) Median OSurv: 10,9 months (dose 9g/sqm) 9-month OSurv: 35% (dose 5g/sqm) 9-month OSurv: 69% (dose 9g/sqm). 12-month OSurv: 19% (dose 5g/sqm) 12-month OSurv: 44% (dose 9g/sqm)	− 23% (dose: 5g/sqm) (6 CR, PR, clinical benefit*) - 36% (dose: 9g/sqm) (13 CR, PR, clinical benefit*)	− 42% (dose: 5g/sqm) (6 CR, PR, clinical benefit* + 5 SD) - 78% (dose: 9g/sqm) (13 CR, PR, clinical benefit* + 15 SD)
Palmerini et al., 2020	51	51	Relapsed and unresectable OS	IFO 3g/sqm/day x 5 days	NR	Median PFS: 6,1 months 4-month PFS: 61% 6-month PFS: 51%	Median OSurv: 14,5 months 1-year OSurv: 62% 2-year OSurv: 30%	− 20% (1 CR + 9 PR)	. 77% (1 CR + 9PR + 29 SD)
Gaspar et al., 2021**	81 pts enrolled and randomized	81	R/R OS	Arm A: Lenvatinib 14 mg/sqm/day x 21 days orally + IFO 3g/sqm/day iv x 3 days + ETOPOSIDE 100 mg/sqm/ day iv x 3 days Arm B: IFO 3g/sqm/day iv x 3 days + ETOPOSIDE 100 mg/sqm/day iv x 3 days	IFO + ETOPOSIDE inpatient	Arm A: 4-month PFS: 76,3% Arm B: 4 month PFS: 66%	Arm A: 12-month OSurv: 49,2% Arm B: 12 month OSurv: 72,1%	Arm A: 15% Arm B: 9,8%	NR
Palmerini et al., 2016	51	40 (35 pts evaluable)	R/R OS	GEMCITABINE: 675–900 mg/sqm oon Day 1 and 8 + DOCETAXEL 75 mg/sqm Day 8.	Outpatient	4-month PFS: 56%	12-month OSurv: 30% (whole cohort)	− 17% (6 PR)	− 57% (6 PR + 14 SD)
Song et al., 2014	28	28 (17 pts evaluable)	R/R OS	GEMCITABINE: 675–900 mg/sqm oon Day 1 and 8 + DOCETAXEL 100 mg/sqm Day 8.	NR	NR	Median OSurv: 9 months 12-month OSurv: 35,3%	− 11,8% (1 CR + 1 PR)	− 41,2% (3CR including 2 metabolic CR + 1 PR + 3 SD)
Navid et al., 2008	22	17 (10 pts evaluable)	R/R OS	GEMCITABINE: 675 mg/sqm oon Day 1 and 8 + DOCETAXEL 75 mg/ sqm Day 8.	Outpatient	NR	NR	− 30% (3 PR)	− 40% (3 PR + 1 SD)
Fox et al., 2012	53	14	Relapsed OS	GEMCITABINE: 675 mg/sqm oon Day 1 and 8 + DOCETAXEL 75 mg/ sqm Day 8.	NR	NR	NR	− 7% (1 PR)	NR
Berger et al., 2009	26	26	Relapsed OS	CYCLOPHOSPHAMIDE: 4 g/sqm on Day 1 + ETOPOSIDE 200 mg/sqm on Days 2, 3, and 4.	Inpatient	4-month PFS: 42%	4-month OSurv: 93% 12-month OSurv: 50%	− 19% (2 CR + 3 PR)	− 54% (2 CR + 3 PR + 9 SD)
Rodriguez-Galindo et al., 2002	14	14	R/R OS	CYCLOPHOSPHAMIDE: 500 mg/sqm/day x 5 days + ETOPOSIDE: 100 mg/sqm/day x 5 days + GCSF	NR	NR	NR	− 28,5% (1 CR + 3 PR)	− 64,2% (1 CR + 3 PR + 5 SD)
Miser et al., 1987	124	17 (8 pts evaluable)	Relapsed OS	IFO 1,8 g/sqm/day x 5 days + ETOPOSIDE: 100 mg/sqm/day x 5 days	Inpatient	NR	NR	− 37,5% (3 PR)	NR
Kung et al., 1993	311	32	R/R OS	IFO: 2 g/sqm x 3 + ETOPOSIDE:100 mg/sqm x 3	NR	NR	NR	− 15,6% (2 CR + 3 PR)	NR

(NR = not reported; OS = Osteosarcoma; R/R = Relapsed/Refractory; pts = patients; PFS = Progression Free Survival; EFS = Event Free Survival; OSurv = Overall Survival; ORR = Overall Response Rate; DCR = Disease Control Rate; CR = Complete Response; PR = Partial Response; SD = Stable Disease. *the authors reported an overall ORR including patients with clinical benefit, not more details are reported; **the results reported in this table for this trial are published on clinicaltrial.gov)

However, previous administration schedules have been associated with significant toxicity, adversely affecting the quality of life of patients with R/R OS [17, 18]. Furthermore the timing of ifosfamide infusion has been shown to correlate with tolerability [20–22].

The administration of high-dose Ifosfamide in a prolonged 14-day continuous infusion using an external pump in an outpatient setting (14-IFO) demonstrated an excellent tolerability and toxicity profile, compared to previously reported schedules (Table 1, Supplementary File) [12, 14–17, 23−30], even among young patients with R/R sarcoma, including bone sarcomas [20]. A prolonged continuous infusion has been shown to be feasible and correlates with reduced incidence of adverse events alongside an improved therapeutic index [20, 31–32].

However, the use of 14-IFO in patients with R/R OS remains limited. This is the first retrospective cohort analysis focused on evaluating the activity and toxicity of 14-IFO within this patient cohort.

## Methods

### Patients and methods

Clinical data from patients diagnosed with OS R/R and treated with 14-IFO were retrospectively analyzed, across five sarcoma centres within the national network of the Italian Paediatric Onco-Haematology Association (AIEOP) and Italian Sarcoma Group (ISG). The following eligibility criteria were required for the present analysis: (i) patients with OS at first or subsequent relapse, defined as the return of the disease following complete surgical tumor remission in either first or subsequent treatment lines or (ii) patients with refractory OS, defined as the persistence of the disease despite surgical and/or chemotherapeutic interventions; (iii) patients aged younger than 40 years at the time of their first dose of 14-IFO; (iv) at least one cycle of 14-IFO received ; (v) radiological tumour assessments according to Response Evaluation Criteria in Solid Tumours (RECIST 1.1) [33].

The primary objective of this trial was to describe the 14-IFO anti-tumour activity in patients with R/R OS, the secondary objective was to assess the treatment’s toxicity profile.

The primary endpoint was PFS, at 4 and 6-months, defined as the ratio between patients achieving Complete Response (CR), Partial Response (PR) or Stable Disease (SD) and those progressing after four and six months from the first dose of 14-IFO. PFS was calculated from the date of the first 14-IFO cycle until either the occurrence of tumour progression or the most recent follow-up. Patients who achieved a surgical complete remission after the treatment with 14-IFO were censored at the time of surgery procedure.

The secondary endpoints were: DCR [defined as the percentage of patients achieving CR + PR + SD], Overall Response Rate (ORR) [defined as CR + PR], both according to RECIST 1.1, and OSurv at 1 and 2-years. OSurv was calculated from the date of the first dose of 14-IFO to the date of death or last follow-up. Patients were censored at the date of last follow-up in the absence of death or progression. Additional secondary endpoint was to assess the toxicity profile of 14-IFO. Treatment-related adverse events were graded according to the National Cancer Institute Common Terminology Criteria for Adverse Events (CTCAE), version 5.0.

An additional exploratory endpoint was the estimation of the growth modulation index (GMI). This index was calculated as the ratio of time to progression with 14-IFO (TTPn) to the most recent prior line of therapy (TTPn-1) for each patient with available progression data prior to 14-IFO initiation. A GMI value of ≥ 1.33 was considered indicative of meaningful clinical activity, as previously defined [38].

Patient and tumour characteristics at initial diagnosis, pattern of recurrence, treatment details, outcome and adverse events were recorded through specific Case Report Forms (CRFs) collected in a retrospective way.

All patients received a total dose of Ifosfamide of 14 g/sqm per cycle, administered over a period of 14 days within a 21-days cycle, mixed with Mesna 14 g/sqm (at a ratio of 1:1), in normal saline solution (total volume up to 275 ml) intravenously via an external pump in an outpatient setting. The external pump was replaced either after 3 or 7 days, depending on local institutional practices. Therefore, patients received Ifosfamide at a dose of 7 g/sqm with Mesna 7 g/sqm during week 1 and week 2 for a total of 14 days or Ifosfamide at a dose of 3 g/sqm with Mesna 3 g/sqm every 3 days for a total of 14 days. No hyperhydration or additional Mesna were administered, but adequate oral hydration (1500 ml/day) was recommended, and antiemetic treatments were provided as needed based on local clinical practices. Antibiotic prophylaxis was not required during chemotherapy infusion and the prophylactic use of G-CSF was not mandatory at the end of chemotherapy infusion, but administered only if deemed necessary.

Clinical and laboratory assessments were performed concurrently with elastomer pump replacement, thus every 3 or 7 days, in accordance with local institutional practices, and one week following the completion of the 14-IFO infusion. Additional assessments were carried out as per local clinical protocols.

Laboratory evaluations comprised full blood count test, liver and kidney function tests, electrolytes and urine analysis. Radiological assessments were scheduled every two or three 14-IFO cycles, following institutional clinical practices, using Computed Tomography scans in adherence to the RECIST criteria version 1.1. Additional radiological assessments were performed as clinically warranted.

The study protocol was approved by the local ethical and regulatory committee of each institute and registered with ClinicalTrials.gov, number NCT04651569. All study procedures were carried out in accordance with the International Council for Harmonisation guidelines on good clinical practice and the STROBE Statement for observational studies.

### Statistical analysis

The distribution of clinical and demographic characteristics of the patients is described using median and inter-quantile ranges for continuous variables and frequencies, and percentages for categorical items. Comparisons of qualitative variables were conducted using the χ² test and Fisher’s exact test as appropriate.

The analysis of OSurv and PFS were conducted using the Kaplan-Meier method with a 95% confidence interval (95% CI). Differences between survival curves were tested through Log-rank tests. The level of statistical significance is set at a value of 0.05. Statistical analyses were performed using R software version 4.2.1.

## Results

Between January 2012, and December 2021, 26 patients with R/R OS were treated with at least one complete 14-IFO cycle in 5 Italian comprehensive sarcoma centres. All patients were evaluable for safety and efficacy. Overall, the mean follow-up period was 16,3 months (range: 5–83).

The median age of patients was 19 years (range: 9–37) at the beginning of 14-IFO; eleven patients (42%) were younger than 18 years old.

Most patients had metastatic disease (23 patients – 88%) and, as expected with OS, metastases were mainly in lungs and bone. Fifteen patients (58%) had refractory disease to two or more previous treatments, whilst the remaining (42%) had relapsed OS.

All patients received the three most common drugs used for treating OS in first line treatment: Methotrexate, Doxorubicin and Cisplatin. Sixteen patients (61%) had previously received Ifosfamide (at a dose of either 10 g/sqm or 15 g/sqm administered over five days) and eight patients (31%) received Mifamurtide during their first-line treatment.

In the subset of patients with relapsed disease, the majority (10/11 patients) relapsed following their first treatment line, with a median disease-free interval (DFI) of 18 months (range: 7.0–33.1) before starting 14-IFO. Only one patient experienced disease relapse after second line treatment with a DFI of 6.8 months before commencing 14-IFO.

Clinical characteristics are described in Table 2.

**Table 2:** Patients’ characteristics:

	*n*°	%
**All**	26	100
**Age median, range (** ***years*** **)**	19 (9–37)	
< 18 years	11	42
≥ 18 years	15	58
**Sex**		
Male	18	69
Female	8	31
**Histological Response for primary tumour at first diagnosis**		
≥ 90%	6	23
< 90%	15	58
Not Available	5	19
**Disease Status at 14-IFO**		
Relapsed OS	11	42
Refractory OS	15	58
**Disease staging at 14-IFO**		
Localized Disease	3 (1 femur, 1 hip, 1 orbit)	12
Metastatic Disease (only lung)	12	46
Metastatic (only bone)	4	15
Metastatic (lung + bone)	2	7,5
Metastatic (lung + other)	3	12
Metastatic (lung + bone + other)	2	7,5
**Disease staging at 14-IFO according to American Joint Commission on Cancer (AJCC) TNM system**		
Stage IIB	2	7,5
Stage III	23	88,5
Data not available	1	4
**Ifosfamide in pre-treatment**		
Yes	16	61
No	10	39
**n° of treatment lines before 14-IFO**		
1 line	13	50
2 lines	5	19
3 lines	5	19
≥ 4 lines	3	12

### Response

**Fig. 1:**
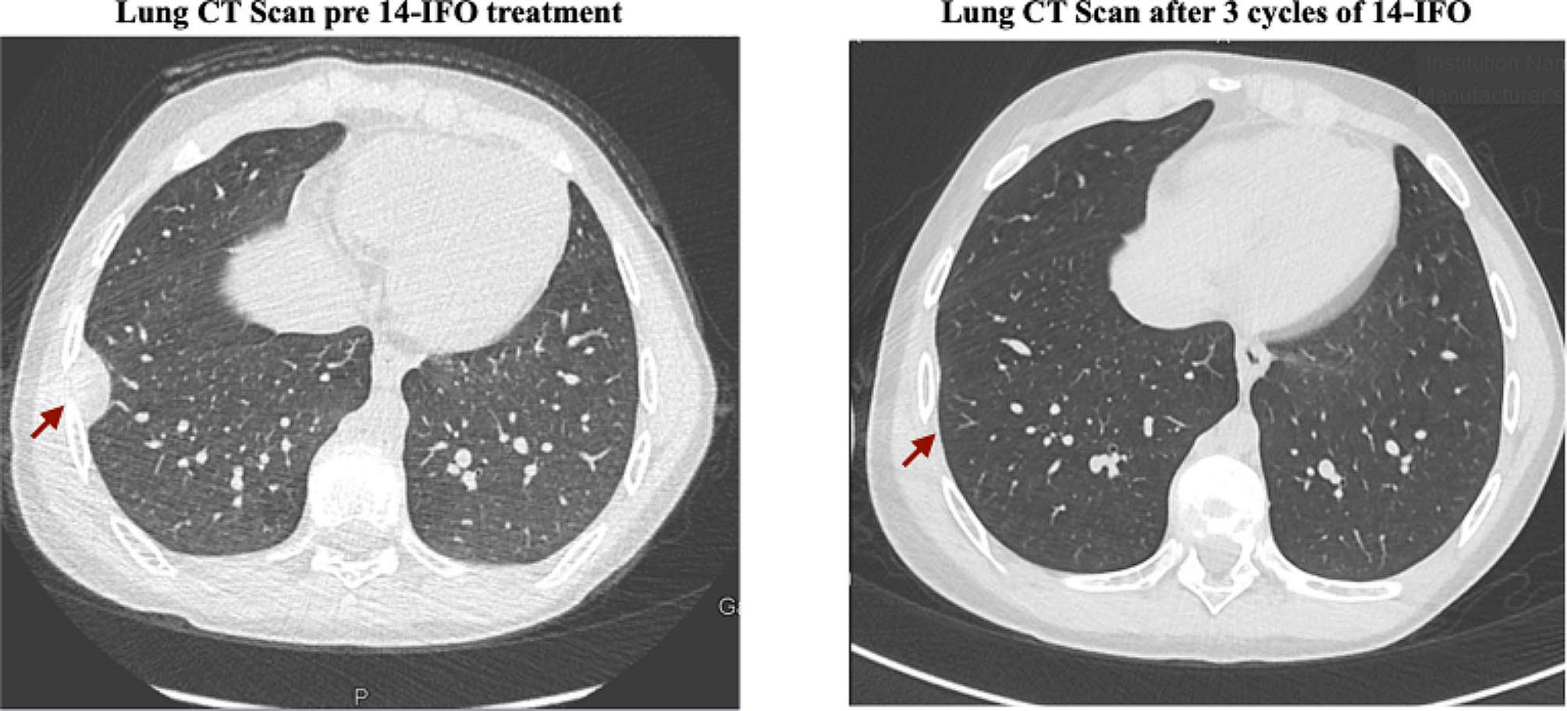
CT Scan images showing complete response according to RECIST Criteria v1.1 in a patient with relapses OS after 3 14-IFO cycles

1 patient achieved a CR (Figs. 1) and 5 patients achieved a PR leading to an ORR of 23%. The median duration of response was 9 months (range: 2–31). ORR was 6.7% and 45% for refractory and relapsed patients, respectively (*p* = 0.054) (Table 3).

**Table 3:** Patient’s characteristic and outcomes according to disease status at the beginning of 14-IFO:

	Refractory pts (tot: 15)	Relapsed pts (tot: 11)	*p*
**Median Age** ***(years), IQR***	21 (17,31)	17 (14, 19)	0.077
**Sex**			> 0.9
Female	5 (33%)	3 (27%)	
Male	10 (67%)	8 (73%)	
**n° of treatment lines before 14-IFO**			0.003
1 line	3 (20%)	10 (91%)	
2 lines	4 (27%)	1 (9%)	
3 lines	5 (33%)	0 (0%)	
≥ 4 lines	3 (20%)	0 (0%)	
**Previous Ifosfamide**			0.10
Yes	11 (73%)	5 (45%)	
No	3 (20%)	6 (55%)	
Data Not Available	1 (6.7%)	0 (0%)	
**Staging at 14-IFO**			0.063
Localized Disease	0 (0%)	3 (27%)	
Metastatic Disease	15 (100%)	8 (73%)	
**n° of 14-IFO cycles received**			0.013
1 cycle	0 (0%)	1 (9%)	
2 cycles	7 (47%)	0 (0%)	
3 cycles	2 (13%)	0 (0%)	
4 cycles	2 (13%)	5 (45,5%)	
≥ 5 cycles	4 (27%)	5 (45,5%)	
**Best Response post 14-IFO**			0.062
Complete Response	0 (0%)	1 (9.1%)	
Partial Response	1 (6.7%)	4 (36%)	
Stable Disease	5 (33%)	4 (36%)	
Progression Disease	9 (60%)	2 (18%)	
**Timing of Best Response achievement**			
Complete Response	/	1 pt after 3 cycles	
Partial Response	1 pt after 4 cycles	2 pts after 2 cycles 2 pts after 4 cycles	
Stable Disease	5 pts after 2 cycles	4 pts after 2 cycles	
**DCR**			0.051
	6 (40%)	9 (82%)	
**ORR**			0.054
	1 (6.7%)	5 (45%)	
**PFS**			0.02
4-month	33%	82%	
6-month	20%	64%	
12-month	0%	18%	
**OSurv**			0.1
4-month	100%	100%	
6-month	93%	91%	
1-year	36%	71%	
2-year	15%	30%	

The DCR was 57.5% for the entire patient cohort (1 CR + 5 PR + 9 SD) and 40% and 82% for refractory and relapsed patients, respectively (*p* = 0.05) (Table 3).

In the cohort of relapsed or refractory patients, no statistically significant differences in DCR or ORR were observed when comparing patients previously treated with High-Dose Ifosfamide (10–15 g/sqm over 5 days) with those who had not received prior treatment with High-Dose Ifosfamide (Table 4).

**Table 4:** Disease Control Rate (DCR) and Overall Response Rate (ORR) in relapsed and refractory patients by previous treatment type. Data about previous treatment with High Dose Ifosfamide were not available for one patien﻿t

Relapsed OS patients			
	Pretreatment with		
	High Dose Ifosfamide		
	No	Yes	P
n	6	5	
ORR (%)	4 (66.7)	1 (20.0)	0.347
DCR (%)	5 (83.3)	4 (80.0)	1
**Refractory OS patients**			
	Pretreatment with		
	High Dose Ifosfamide		
	No	Yes	p
n	3	11	
ORR (%)	1 (33.3)	0 (0.0)	0.47
DCR (%)	2 (66.7)	3 (27.3)	0.56

For seven patients (27%), local treatment was feasible following 14-IFO administration. Specifically, five patients underwent surgery, including two patients who underwent bilateral thoracic procedures (one individual at different times following the second and third cycles, and one individual after the fourth cycle); two patients who underwent lateral thoracic procedures after 4 and 8 cycles, respectively; one patient who underwent a local maxillary procedure after 5 cycles. One patient received local Carbon-Ion radiotherapy for vertebral metastases after 4 cycles, and another patient underwent photon-radiotherapy for vertebral metastases after 4 cycles.

Notably, one patient with a localised orbital OS achieved a PR according to RECIST 1.1, and a complete metabolic response confirmed by Positron Emission Tomography/Computed Tomography (PET-CT) Total Body after 4 cycles. Subsequently, the patient underwent local surgery, achieving a macroscopic CR.

GMI was assessable for 13 patients (all of them in the relapsed OS cohort). Only one patient showed a GMI greater than 1.33.

### Survival

**Fig. 2:**
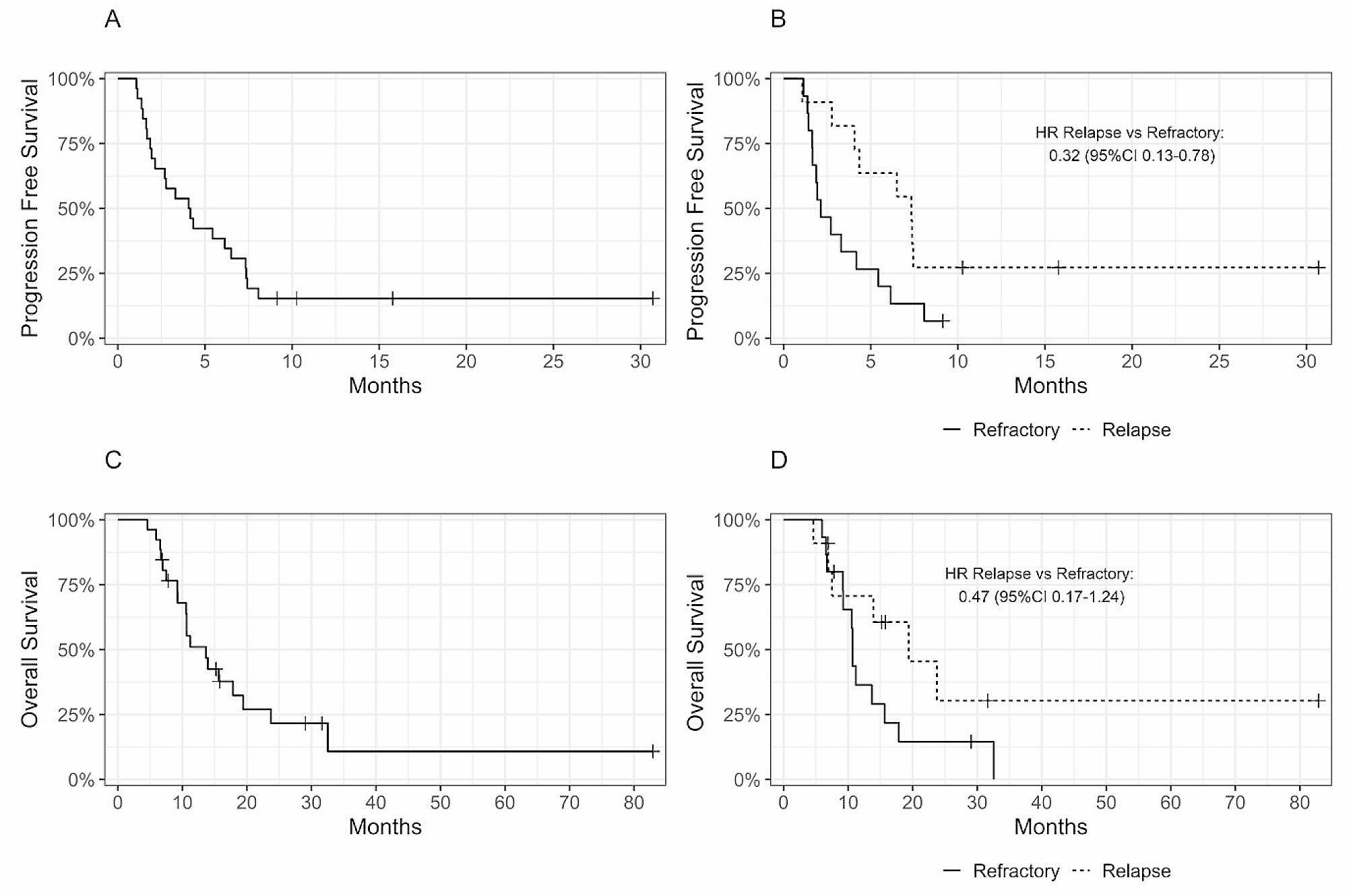
Kaplan-Meier curve for Progression Free Survival (**A**), Progression Free Survival according to status pre 14-IFO (**B**), Overall Survival (**C**), Overall Survival according to status pre 14-IFO (**D**)

The median PFS was 4.1 months [95% CI 2.13, 7.37] for the whole cohort. Four-month and 6-month PFS were 54% [95% CI 38–77] and 38% [95% CI 24–63], respectively (Fig. 2A). PFS was significantly better in the group of patients with relapsed OS compared to patients with refractory disease (HR: 0.32, [95% CI 0.13, 0.78]). Median PFS was 7.33 months for relapsed patients vs. 2.13 months for refractory patients (*p* = 0.02). Four and 6-month PFS were 33% [95% CI 16–68] and 20% [95% CI 7.33-55], respectively, for refractory patients compared to 82% [95% CI 62–100] and 64% [95% CI 41–99] for relapsed patients. (*p* = 0.02) (Fig. 2B).

Moreover, patients younger than 18 years old had a higher PFS rate compared to older patients. Four- and 6-month PFS were both 73% [95% CI 51–100] for patients younger than 18 years old at the beginning of 14-IFO treatment, compared to 40% [95% CI 22–74] and 13% [95% CI 3.7–48] for patients older than 18 (HR: 2.87 95% CI (1.13–7.29), *p* = 0.03).

The median OSurv was 13.7 months [95% CI 10.6–23.7] for the whole cohort. One-year and 2-year OSurv were 51% [95% CI 35–75] and 22% [95% CI 9.5–49], respectively (Fig. 2C).

Median OSurv was longer for relapsed patients than refractory patients (19.4 vs. 10.7 months (*p* = 0.1). One-year OSurv was 71% [95% CI 48–100] *and* 36% [95% CI 18–73], for relapsed and refractory patients, respectively. Meanwhile, 2-year OSurv was 30% [95% CI 10–91] vs. 15% [95% CI 4–52], for relapsed and refractory patients, respectively (*p* = 0.1) (Fig. 2D).

No significant PFS and OSurv differences were observed according to sex, staging, previous histological response, and previous treatments. More interestingly, no statistically significant PFS and OSurv differences were detected comparing patients previously treated with High Dose Ifosfamide (10–15 g/sqm over 5 days) with patients without a prior treatment with High Dose Ifosfamide.

Six-month PFS was 56% [95% CI 31–100] for patients who didn’t receive High Dose Ifosfamide in a previous line vs. 25% [95% CI 11–58] for patients pretreated with High Dose Ifosfamide (Log-rank *p* = 0,2).

One- and 2-year OSurv were 53% [95% CI 28–100] and 27% [95% CI 8.3–86], respectively, for patients who didn’t receive High Dose Ifosfamide in a previous line vs. 54% [95% CI 34–86] and 20% [95% CI 6.1–63], for patients pretreated with High Dose Ifosfamide (Log-rank *p* = 0,7).

### Treatment administration and toxicities

Treatment administration, with details regarding treatment delay, dose reductions and toxicities, is presented in Table 5.

**Table 5:** 14-IFO cycles administration and toxicities:

	*n*°	%
**All patients**	26	100
**Cycles received**		
1 cycle	1	4
2 cycles	7	27
3 cycles	2	8
4 cycles	7	27
5 cycles	4	15
6 cycles	4	15
8 cycles	1	4
**Delayed cycle**		
Yes (toxicities reason)	6	23
Yes (organizational reason)	1	4
No	19	73
**n° of delayed cycles/patient**		
1 cycle	4	15
2 cycles	1	4
3 cycles	1	4
4 cycles	1	4
**Dose Reduction**		
Yes	5	19
No	21	81
	**n°**	**%**
**All Cycles**	**101**	100
**Leukopenia g.1–2**	41	40.5
**Leukopenia g.3**	18	19
**Leukopenia g.4**	6	5.8
**Neutropenia g.1–2**	37	36.6
**Neutropenia g.3**	15	14.8
**Neutropenia g.4**	9	8.9
**Thrombocytopenia g.1–2**	16	15.8
**Thrombocytopenia g.3**	3	2.9
**Thrombocytopenia g.4**	1	0.9
**Febrile Neutropenia g.3**	1	0.9
**Febrile Neutropenia g.4**	0	0
**Nausea/Vomiting g.1–2**	4	3.9
**Nausea/Vomiting g.3**	0	0
**Nausea/Vomiting g.4**	0	0
**Fatigue g.1–2**	3	2.9
**Fatigue g.3**	0	0
**Fatigue g.4**	0	0
**Neurological g.1–2**	0	0
**Neurological g.3**	0	0
**Neurological g.4**	0	0
**Renal g.1–2**	0	0
**Renal g.3**	2*	1,9
**Renal g.4**	0	0

*1 patient started 14-IFO treatment with an ongoing renal toxicity g.2 and during the 2nd course exhibited a worsening of the pre-existing toxicity up to g.3 (creatinine and eGFR increased)

Most patients (sixteen patients – 61%) received at least four 14-IFO cycles. A total of 101 cycles were administered and all evaluable for toxicity assessment.

No patient permanently discontinued the treatment because of adverse events. Treatment delay occasionally occurred in 7 (27%) out of 26 patients (6 patients for toxicity and 1 patient due to logistic reasons), however, most of these delays involved only one cycle throughout the entire treatment period.

Dose reductions were reported in five (19%) patients. These reductions were primarily attributed to adverse events, particularly hematological toxicities. Specifically, one patient received four cycles with a 50% dose reduction, three patients received one cycle each with a 25% dose reduction (equivalent to 75% of the full dose), and one patient received one cycle with a 15% dose reduction (equivalent to 85% of the full dose).

Overall, the most common grade 3 or worse treatment-related adverse events included haematological toxicities. Grade 3–4 haematological events were observed in 53 cycles (52%) as follows: (i) white-blood cell decrease in 24 cycles (23.7%); (ii) neutropenia in 24 cycles (23.7%); (iii) thrombocytopenia in 4 cycles (3.9%).

In addition, 12 patients (46%) experienced grade 3–4 leukopenia during at least one 14-IFO cycle, with 11 of them concurrently experiencing grade 3–4 neutropenia (42%). However, only five patients required G-CSF administration resulting in rapid blood count recovery. Two patients (7.6%) showed grade 3–4 thrombocytopenia in at least one cycle and only one patient (3.8%) experienced an episode of febrile neutropenia, requiring hospital admission. Otherwise, no other admissions related to 14-IFO treatment were reported. No grade 3–4 anaemia or non-haematological toxicities were reported. Moreover, throughout the whole treatment period, no patients exhibited neurological toxicities. Notably, one patient experienced deterioration of a pre-existing renal toxicity. This patient commenced 14-IFO treatment with an ongoing grade 2 renal toxicity according to CTCAE v.5.0 (chronic kidney disease with increased creatinine and reduced estimated Glomerular Filtration Rate [eGFR]) and after two 14-IFO cycles, the renal toxicity worsened compared to baseline, reaching grade 3.

Previous treatment with High-Dose Ifosfamide (10 g/sqm or 15 g/sqm delivered over five days infusion) did not increase the toxicity incidence rate, nor affected haematological and non-haematological toxicities.

Patients and their caregiver did not encountered difficulties in managing the elastomer-pump at home, and no patients required telehealth assistance or face-to-face support due to technical pump issues.

## Discussion

Our results provided evidence of the anti-tumour activity of 14-IFO in heavily pre-treated paediatric and young adult patients with R/R OS, alongside a good quality of life.

The ORR and DCR obtained with 14-IFO was 23% and 57.5%, respectively. Notably, patients with relapsed OS showed a higher ORR (45%) and DCR (82%) compared to refractory patients, irrespective of the number of prior treatment lines received. The achievement of disease control with 14-IFO administration allowed 27% of patients to undergo subsequent local treatment, which is still considered the best treatment option for R/R OS [10]. For 71% of them, the local treatment was a new surgical procedure.

Four-month PFS was 54% for the whole cohort of patients and 82% for the relapsed OS sub-group, highlighting a positive outcome for patients with advanced OS according to the recent clinical trials outcomes recommendation [8]. Median OSurv was 13.7 months and 1-year OSurv was 51% for all patients and 71% for relapsed patients. Aside from age and disease status, where an age over 18 years old and refractory disease status were identified as negative prognostic factors, other clinical features did not significantly influence the outcome. Furthermore, no significant PFS and OSurv differences were observed according to previous treatment regimens. More interestingly, our analysis revealed that prior treatment with High-Dose Ifosfamide (10 g/sqm or 15 g/sqm delivered over five days infusion) did not influence the survival rate or the incidence of toxicity. While these findings warrant confirmation in a larger patient cohort, they suggest that 14-IFO could be a viable and well-tolerated therapeutic option also for patients with R/R OS previously treated with High-Dose Ifosfamide.

Compared to other chemotherapy or tyrosine kinase inhibitor regimens recommended by major International OS Guidelines [1, 34], this trial demonstrated that the14-IFO schedule is not only feasible but also exhibits a significant antitumour activity in this setting. While previously published results for R/R OS encompass a heterogeneous patient population, and randomized controlled trials are infrequent in this setting, the outcomes described here align with and are comparable to those reported in the literature including High-Dose Ifosfamide administered at a schedule of 3 g/sqm over 5 days [12]. In particular, in our analysis, we observed an ORR of 20%, consistent with the previously reported ORR of 23% with the use of High-Dose Ifosfamide delivered over 5 days [12]. Furthermore, in our analysis the 2-year OSurv was 22% for the overall cohort and 30% for the relapsed OS subgroup, compared to the previously reported 2-year OSurv of 30% for the different schedule infusion [12]. It is noteworthy that our cohort was more heavily pretreated, with 50% of patients having received more than one treatment before 14-IFO, compared to only 10% of patients in the previous study using the High dose Ifosfamide schedule over 5 days [12].

Regarding the GMI data, it is important to highlight that our GMI evaluation remains exploratory and is limited by the small number of patients assessed for this analysis. In a context where further studies are required to confirm the overall reliability of GMI in assessing the efficacy of experimental drugs in advanced OS [39], our findings contribute to this ongoing investigation.

Considerably, our results highlight the manageable toxic profile of the 14-IFO regimen. Patients were able to receive chemotherapy treatment at home with scheduled clinical visits (every 3 or 7 days, according to local practice) using an external elastomer-pump carefully charged with the optimal Ifosfamide dose and MESNA.

Patients treated with 14-IFO showed a tolerable toxic profile in terms of haematological and non-haematological events (including nausea, gastrointestinal, renal and neurological events). Ifosfamide-induced encephalopathy, although rare, is a well-known adverse event which can occur in 2–5% of patients who have received the drug either intravenous or orally [36, 37] It can occur 12 to 146 h after the start of the infusion with different clinical features (from mild to life-threatening symptoms) [36, 37]. Notably, the cohort of patients analyzed in this study didn’t show any neurological toxicities, consistent with the previously reported association with continuous infusion and fewer side-effects, including low incidence of neurological events [19–22]. In fact, a prolonged infusion induces a reduction in the half-life of the active metabolites of ifosfamide, increasing their clearance and giving rise to a lower plasma peak, without changing the drug’s alkylating activity [19–22].

Due to the low toxic profile, patients treated with 14-IFO, could pursue their routinely activities outside of hospital, preserving their quality of life. The daily and nocturnal management of elastomer pump was feasible at home with a little bag or backpack or in a large pyjama trouser pocket, without any unexpected technical issues at home.

In the context of a rare tumour with a poor prognosis, it is strongly encouraged that the treatment options are guided by a careful balance between the potential for cure, the toxicity profile of the intended treatment, and the patient’s quality of life [2, 35]. For this reason, therapeutic decisions should prioritize the least toxic treatment option, and 14-IFO should be regarded as a viable course of action. Our results suggest that the use of 14-IFO represents a promising alternative option to other chemotherapy regimen, especially for Relapsed OS patients. Patients with Refractory OS continue to represent an unmet clinical need, highlighting the necessity for further research efforts to enhance our understanding and counteract the aggressive biological behavior of this disease.

### Electronic supplementary material

Below is the link to the electronic supplementary material.

Supplementary Material 1

## Data Availability

Anonymized data that support the findings of this study are available from the corresponding author, AC and VS, upon request.
