# Supplementary material for: Prolonged 14-day continuous infusion of high-dose ifosfamide for patients with relapsed and refractory high-grade osteosarcoma: a retrospective multicentre cohort study
Source: BMC Cancer. 2024 Jun 19;24:747. doi: 10.1186/s12885-024-12498-x (PMC11186082; doi:10.1186/s12885-024-12498-x)
Supplement: Supplementary file 1 — Supplementary Material 1 [file 12885_2024_12498_MOESM1_ESM.pdf]

Supplementary - Table 1: toxicities reported in previous published chemotherapy regimens for patients with R/R OS

	Drugs and Schedule	Toxicities reported
**Harris et al., 1995**	IFO: 2,400 mg/sqm/day x 5 days	Stratum 1: Severe white blood cells decreased: 18 courses, Life-Threatening white blood cells decreased: 17 courses, Severe Neutropenia: 6 courses, Life-Threatening Neutropenia: 30 courses, Life-Threatening Platelets decreased: 1 course, Severe Bacterial Infection: 1 course. Stratum 2: Severe white blood cells decreased: 8 courses, Life-Threatening white blood cells decreased: 33 courses, Severe Neutropenia: 3 courses, Life-Threatening Neutropenia: 38 courses, Severe Platelets decreased: 4 courses, Life-Threatening Platelets decreased: 4 courses, Severe Hemoglobin decreased: 7 courses, Severe Hypokalemia: 2 courses, Life-Threatening Hypokalemia: 1 course, Severe creatinine increased: 1 course.
**Berrak et al., 2005**	IFO: 2 g/sqm/dose administered intravenously at 12 hr interval x 7 days = total dose 14g/sqm	Neutropenia and fever: 12/84 courses; - Neutropenia and fever with admission: 5 patients (31,2%); - Renal toxicity: 4 patients (25%)(2 patients: elevated levels of serum creatinine; 2 patients: full-blown chronic renal failure); - Hemorrhagic cystitis: 1 patients x 3 courses;
**Patel et al., 1997**	IFO: 14 g/sqm (bolus or continuos infusion)	**Phase II (n=311 courses):** Dose level 14 g/sqm: neutropenia, grade 3-4:147 courses (74%), febrile neutropenia: 54 courses (27%), thrombocytopenia, grade 3-4: 60 course (30%), anemia, grade 3-4: 61 courses (31%), renal, grade 3-4: 2 courses (1%), renal, grade 1-2: 18 courses (9%), neurological events, grade 3: 2 courses (1%), neurological events, grade 1-2: 105 courses (53%), cardiac, grade 3: 1 course (0,5%), hematuria, grade 3: 3 courses (1,5%), vomiting, grade 3: 5 courses (2,5%), peripheral neuropathy, grade 3: 2 courses (1%). Dose level 12 g/sqm: neutropenia, grade 3-4: 40 courses (82%), febrile neutropenia: 13 courses (27%), thrombocytopenia, grade 3-4: 25 courses (51%), anemia, grade 3-4: 32 courses (65%), renal, grade 3-4: 0 course, renal, grade 1-2: 8 courses (16%), neurological events, grade 3: 1 course (2%), neurological events: grade 1-2, 29 courses (59%), cardiac, grade 3: 0 course, hematuria, grade 3: 3 courses (6%), vomiting, grade 3: 2 courses (4%), peripheral neuropathy, grade 3: 0 course. Dose level 10 g/sqm: neutropenia, grade 3-4: 47 courses (73%), febrile neutropenia: 16 courses (25%), thrombocytopenia, grade 3-4: 36 courses (56%), anemia, grade 3-4: NR, renal, grade 3-4: 0 course, renal, grade 1-2: 12 courses (19%), neurological events, grade 3: 0 course, neurological events, grade 1-2: 26 courses (41%), cardiac, grade 3: 0 course, hematuria, grade 3: 1 course (1,5%), vomiting, grade 3: 0 course, peripheral neuropathy, grade 3: 0 course. **Pilot Phase (n=48 courses):** Dose level 14 g/sqm: neutropenia, grade 3-4: 32 courses (78%), febrile neutropenia: 14 courses (29%), thrombocytopenia, grade 3-4: 11 courses (27%), anemia, grade 3-4: 11 courses (27%), renal, grade 3-4: 0 course, renal, grade 1-2: 13 courses (32%), neurological events, grade 3: 0 course, neurological events, grade 1-2: 7 courses (17%), cardiac, grade 3: 0 course, hematuria, grade 3: 0 course, vomiting, grade 3: 0 course, peripheral neuropathy, grade 3: 0 course. Dose level 12 g/sqm: neutropenia, grade 3-4: 1 course (20%), febrile neutropenia: 0 course, thrombocytopenia, grade 3-4: 1 course (20%), anemia, grade 3-4: 3 courses (60%), renal, grade 3-4: 0 course, renal, grade 1-2: 2 courses (40%), neurological events, grade 3: 0 course, neurological events, grade 1-2: 2 courses (20%), cardiac, grade 3: 0 course, hematuria, grade 3: 0 course, vomiting, grade 3: 0 course, peripheral neuropathy, grade 3: 0 course. Dose level 10 g/sqm: neutropenia, grade 3-4: 1 course (50%), febrile neutropenia: 0 course, thrombocytopenia, grade 3-4: 1 course (50%), anemia, grade 3-4: 1 course (50%), renal, grade 3-4: 0 course, renal, grade 1-2: 0 course, neurological events, grade 3: 0 course, neurological events, grade 1-2: 0 course, cardiac, grade 3: 0 course, hematuria, grade 3: 0 course, vomiting, grade 3: 0 course, peripheral neuropathy, grade 3: 0 course.
**Verschoor et al., 2020**	IFO 5 g/sqm as bolus infusion (26 patients) or IFO 3 g/sqm x 3 days continuos (36 patients)	Dose < 6 g/sqm: febrile neutropenia 4%, neutropenia 12%, vomiting 12%, syncope 4% Dose > 6g/sqm: febrile neutropenia 19%, neutropenia 19%, anemia 8%, nausea 14%, encephalopathy 8%, hypophosphatemia 3%, constipation 3%, mucositis 3%, anorexia 3%, acute kidney injury 6%, dehydration 3%
**Palmerini et al., 2020**	IFO 3g/sqm/day x 5 days	Febrile neutropenia: 9 (18%) patients, Anemia grade 3-4: 2 (4%) patients, Thrombocytopenia: 3 (6%) patients, Neurological toxicity grade 3-4: 2 (4%) patients, Kidney injury: 2 (4%) patients.
**Palmerini et al., 2016**	GEMCITABINE: 675–900 mg/sqm oon Day 1 and 8 + DOCETAXEL 75 mg/sqm Day 8.	Toxicities reported for the whole cohort: g. 4 haematological toxicities: 13 patients (25%), g.4 neutropenia: 11 patients (21%), g.4 thrombocytopenia: 2 patients (3,9%), allergic reactions: 3 patients (6%).
**Song et al., 2014**	GEMCITABINE: 675–900 mg/sqm oon Day 1 and 8 + DOCETAXEL 100 mg/sqm Day 8.	g.3 anemia: 1 patient (3,6%), g.3 Neutropenia: 3 patients (10,7%, g.4 Neutropenia: 4 patients (14,2%), g.3 Thrombocytopenia: 4 patients (14,3%), g.4 Thrombocytopenia: 2 patients (7,1%), g.3 Febrile Neutropenia: 1 patient (3,6%), g.4 Pneumonitis: 1 patient (3,6%), g.3 palmar-plantar erythrodysesthesia: 2 patients (7,1%).
**Navid et al., 2008**	GEMCITABINE: 675 mg/sqm oon Day 1 and 8 + DOCETAXEL 75 mg/sqm Day 8.	Toxicities reported for the whole cohort: g.3 Anemia: 7 patients (32%), g.4 Anemia: 2 patients (9%), g.3 Neutropniea: 11 patients (50%), g.4 Neutropenia: 8 patients (36%), g.3 Thrombocytopenia: 9 patients (41%), g.4 Thrombocytopenia: 9 patients (41%), g.3 Diarrhea: 2 patients (9%), g.3 Nausea: 1 patient (5%), g.3 Vomiting: 1 patients (5%), g.3 Colitis: 1 patient (5%), g.3 elvated ALT/AST: 3 patients (14%), g.3 Infection without neutropenia: 3 patients (14%), g.3 Infection with neutropenia: 1 patient (5%), g.3 Sensory neuropathy: 1 patient (5%), g.3 Fluid retention: 2 patients (9%).
**Fox et al., 2012**	GEMCITABINE: 675 mg/sqm oon Day 1 and 8 + DOCETAXEL 75 mg/sqm Day 8.	Toxicities reported for the whole cohort: Neutropenia, thrombocytopenia, fatigue, dyspnea, bronchospasm, edema, neuropathy, liver function anmormalities.
**Berger et al., 2009**	CYCLOPHOSPHAMIDE: 4 g/sqm on Day 1 + ETOPOSIDE 200 mg/sqm on Days 2, 3, and 4.	g.4 hematological toxicities: all patients (100%), g.3 Fever: 9 courses (16%), g.4 Fever: 3 courses (5%), g.3 mucositis: 4 courses (7%), g.4 mucositis: 10 courses (18%),
**Rodriguez-Galindo et al., 2002**	CYCLOPHOSPHAMIDE: 500 mg/sqm/day x 5 days + ETOPOSIDE: 100 mg/sqm/day x 5 days + GCSF	Only 13 of the 47 courses were evaluable for toxicities. g.4 Neu- tropenia: 12 courses (92.3%). All the patients required transfusions of packed red blood cells and platelets. Complications resulting from infection with fever and neutropenia: 9 instances, central-line infection: 2 instances.
**Miser et al., 1987**	IFO 1,8 g/sqm/day x 5 days + ETOPOSIDE: 100 mg/sqm/day x 5 days	Toxicities reported for the whole cohort: g.3-4 Neutropenia: 97% of total courses, Fever: 33% of the total courses, g.3-4 Thrombocytopenia: 32% of the total courses, g.3-4 Nausea or Vomiting: 13% of the total courses, Sepsis: 7% of the total courses, g.3-4 of Neurologic Toxicity: 2% of the totale courses, g.3-4 Hepatotoxicity: 4% of the total courses.
**Kung et al., 1993**	IFO: 2 g/sqm x 3 + ETOPOSIDE: 100 mg/sqm x 3	Toxicities reported for the whole cohort: Neutrophils lower than 1000/microl: 81% of pts, Vomiting: 45% of pts, Platelets lower than 50.000/microl: 25,5% of pts, Bacterial Infection: 34% of pts, Hematuria, microscopic: 18,5% of pts, Neurotoxicity: 5,8% of pts, Hematuria, gross: 4% of pts.
